# Effect of nasal carriage of *Bacillus* species on COVID-19 severity: a cross-sectional study

**DOI:** 10.1128/spectrum.01843-23

**Published:** 2024-01-09

**Authors:** Muinah A. Fowora, Adenike Aiyedogbon, Ibilola Omolopo, Ahmed O. Tajudeen, Abdul-Lateef Olanlege, Adefunke Abioye, Grace B. Akintunde, Babatunde L. Salako

**Affiliations:** 1 Molecular Biology and Biotechnology Department, Nigerian Institute of Medical Research, Yaba, Lagos, Nigeria; 2 Department of Science Laboratory Technology, Faculty of Science., Lagos State University, Ojo, Lagos, Nigeria; 3 Infectious Diseases Hospital, Lagos, Nigeria; Suranaree University of Technology, Meung, Nakhon Ratchasima, Thailand

**Keywords:** COVID-19, *Bacillus* species, nasal spray, severity

## Abstract

Intranasal sprays containing *Bacillus* species are being researched for treating viral respiratory tract infections. The aim of this study was to assess the relationship between the nasal carriage of *Bacillus* and COVID-19 severity. This was a cross-sectional study that collected nasopharyngeal samples from adults 18 years and above visiting two COVID-19 testing centers in Lagos, Nigeria, between September 2020 and September 2021. *Bacillus* species were cultured from the samples and confirmed using 16 s rRNA gene sequencing. The dependent variable was COVID-19 status classified as negative, asymptomatic, mild, or severe. The independent variable was the nasal carriage of *Bacillus* species. Multinomial regression analysis was done to determine the association between nasal carriage of *Bacillus* and COVID-19 severity after adjusting for age, sex, and co-morbidity status. A total of 388 participants were included in the study with mean (standard deviation) age of 40.05 (13.563) years. Sixty-one percent of the participants were male, 100 (25.8%) had severe COVID-19, 130 (33.5%) had pre-existing comorbidity, and 76 (19.6%) had *Bacillus* cultured from their nasopharyngeal specimen. *Bacillus* species presence was significantly associated with higher odds of severe COVID-19 compared to having a negative COVID-19 status (AOR = 3.347, 95% CI: 1.359, 8.243). However, the presence of *Bacillus* species was significantly associated with lower odds of severe COVID-19 compared to having a mild COVID-19 status. The study suggests that nasal carriage of *Bacillus* species is associated with the clinical course of COVID-19 and supports the exploration of *Bacillus* species in the management of viral respiratory tract infections.

**IMPORTANCE:**

With the introduction of intranasal spray containing *Bacillus* species for the treatment of viral respiratory tract infections, such as COVID-19 and respiratory syncytial virus, identifying the association between the nasal carriage of *Bacillus* species and COVID-19 susceptibility and severity will help further substantiate the investigation of these bacteria for COVID-19 prevention and treatment. This study evaluated the association between the carriage of *Bacillus* species in the nasopharyngeal tract and COVID-19 severity and found that the presence of *Bacillus* species in the nasopharynx may significantly impact the clinical course of COVID-19.

## INTRODUCTION

Corona Virus Disease 2019 (COVID-19), an acute and highly infectious respiratory disease caused by the severe acute respiratory syndrome coronavirus 2 (SARS-CoV-2), has led to the death of more than 6.42 million people globally since its outbreak in Wuhan, China, in 2019 (1). Several factors such as the nasal microbiota determine the susceptibility and severity of the disease (2–7). Microbiota can confer some level of protection on the host against some diseases by creating a unique microbial ecosystem that enhances resistance against the manifestation of respiratory tract infection caused by bacterial, fungal, and/or viral pathogens (8, 9). It also serves as markers of disease (10) and the regulation of local and systemic immunity in the nasopharyngeal tract, thereby influencing COVID-19 susceptibility and clinical outcome (7). In addition, viral infection modifies the host’s nasal microbiota, decreases the nasal and gut microbiota diversity, and increases the disease infectivity and severity (11, 12).

A significant increase in bacteria belonging to the phyla Bacteroides, Proteobacteria, Actinobacteria, Firmicutes, and Fusobacteria has been reported among COVID-19 patients (13) though some other study did not find a significant difference in the nasal bacterial composition and diversity in patients with COVID-19 when compared with patients negative for COVID-19 (14). The members of bacteria belonging to the phylum Firmicutes, especially of the class Bacilli, have been associated with respiratory immunity and strengthening the host defense against respiratory viral infections such as influenza, Coronavirus, and respiratory syncytial virus (11, 15). Some of the ways *Bacillus* can strengthen the host defense against viral infection is through the production of surfactants which can reduce the entry of respiratory viruses into the epithelial cells (15, 16). *Bacillus* has been explored as intranasal sprays for the treatment of viral infection (17, 18) as it eases the symptoms of acute respiratory tract infection caused by respiratory syncytial virus by reducing the viral load and inflammation associated with the disease (18). *Bacillus subtilis* possesses the probiotic potential to increase mucosal and tonsillar immunity against respiratory diseases through the formation of immune cells in the nasal cavity and tonsil (17). Like bacilli intranasal sprays, lactobacilli-containing throat sprays have also been demonstrated to considerably lessen the acute symptoms of COVID-19 as well as the load of the virus in the nasopharynx (19). However, there are currently no studies showing the effect of nasal carriage of *Bacillus* species on the susceptibility to SARS-COV-2 infection and severity.

The aim of this study was to determine the association between nasal carriage of *Bacillus species* and COVID-19 susceptibility and severity. We hypothesize that there will be a negative association between nasal carriage of *Bacillus* and COVID-19 susceptibility and severity.

## MATERIALS AND METHODS

### Study design and population

This study was a cross-sectional study that compared the prevalence of nasal carriage of *Bacillus* species among adults who were tested positive or negative for COVID-19 in Lagos State, Nigeria. The study population included individuals ≥ 18 years who presented for SARS-CoV-2 testing at the COVID-19 modified drive-through centre of the Nigerian Institute of Medical Research and the laboratory of the Infectious Disease Hospital, Lagos. The participants were recruited between September 2020 and September 2021. Adults who had traveled out of the country in the last month of the survey were excluded from the study. The COVID-19 vaccine was not available in Nigeria at the commencement of this study. Subsequently, participants who were vaccinated were excluded from the study. Informed consents were appropriately obtained from all eligible participants.

### Sample size and sampling technique

The sample size for this study was determined using the formula for sample size calculations for prevalence studies (20). The standard normal variate was set at 5% type I error; the precision was set at 0.05; and the expected proportion of adults with nasal *Bacillus* was set at 50% in the absence of any available data. The minimum sample size was 385. The sample size was increased to 450 participants to account for missing data or invalid COVID-19 results. A convenient sample of participants willing to participate in the study was recruited.

### Data collection

Information on the age (age as at last birthday) and sex (sex at birth, male/female) of participants were collected. Other information collected were the symptoms and severity of the patient and if the participant had any underlying conditions such as diabetes, asthma, hypertension, heart, lung, and kidney disease. These variables have been identified factors that influence COVID-19 susceptibility and severity (21, 22). No identifying information was collected from the participants.

Participants were classified as positive for COVID-19 when the results on the quantitative real-time reverse transcription-polymerase-chain-reaction (qRT-PCR) assay of their nasopharyngeal swab specimens were positive. Participants with positive test results were categorized as asymptomatic, mild, or severe cases using the guidelines of the Nigeria Centre for Disease Control and the National Institute of Health (23, 24). Asymptomatic cases were defined as patients that had no symptoms specific to COVID-19 but tested positive for SARS-CoV-2. Mild cases were patients who had symptoms corresponding to COVID-19 such as fever (>38°C), cough, sore throat, malaise, headache, muscle pain, loss of taste and smell, nausea, vomiting, diarrhea, abdominal pain, oxygen saturation measured by pulse oximetry (SpO_2_) value ≥94%, shortness of breath but do not have difficulty breathing, and any underlying conditions. Severe cases were patients with clinical manifestations of COVID-19 in conjunction with difficulty in breathing, reduced/decreased breath sounds, SpO_2_ value <94%, a respiratory rate >30 breaths/min, and presence of an underlying comorbid condition such as diabetes, asthma, hypertension, heart, lung, asthma, and kidney disease.

### Sample collection and analysis

Nasopharyngeal swab samples were collected from the participants into two different transport media. One containing a viral transport medium (VTM) and the other a bacterial transport medium containing Tryptone soy broth buffered with 2.5% saline and 10% glycerol. All samples were analyzed within 2 h of specimen collection.

#### RNA extraction and qRT-PCR

Viral nucleic acid extraction was carried out on the nasopharyngeal sample in VTM using the Qiagen Viral Extraction kit according to manufacturer’s instructions, and SARS‐CoV‐2 was detected by quantitative reverse transcription polymerase chain reaction (qRT‐PCR) using a commercial Nucleic Acid Diagnostic Kit for COVID-19, Sansure Novel Coronavirus (2019-nCoV), according to manufacturer’s protocol (Sansure Biotech Inc). The Sansure kit has high sensitivity of about 95.3% and is endorsed for SARS-COV-2 testing (25).

#### Bacteria isolation

The nasopharyngeal samples in the bacteria transport medium were plated on Mueller Hinton Agar (Oxoid, USA), a *Bacillus* ChromoSelect Agar (Merck, Germany), which is selective and differential for *Bacillus* species, using a disposable loop. The agar plates were incubated aerobically at 37°C for 24 h.

#### Bacterial identification

Presumptive identification of the isolates was carried out using colony morphology and biochemical identification. Isolates on Mueller Hinton agar that appeared flat or convex with irregular edges, with a dry-wrinkled colony, and have a sticky consistency when picked with a loop were considered as *Bacillus* species. The presence of growth on the corresponding plate of the *Bacillus* ChromoSelect Agar was further used as an indication of *Bacillus* growth. The isolates were further identified using Gram staining, nitrate reduction, gelatin hydrolysis, and citrate utilization as previously described (26, 27).

The isolates were confirmed as *Bacillus* species using Polymerase Chain reaction (PCR) and 16S rRNA gene sequencing. Deoxyribonucleic acid (DNA) was extracted from all the presumptively identified bacteria using the NIMR Biotech DNA extraction kit. To confirm the identification of the *Bacillus* species, polymerase chain reaction amplification of the 16 s rRNA gene specific for *Bacillus* was carried out. The reaction was done using the primer set 16S-HV (Forward primer-5′-GCCTAATACATGCAAGTCGAGCG-3′ and Reverse primer-5′- ACTGCTGCCTCCCGTAGGAGT-3′) as previously described (28).

Polymerase chain reaction was done using the Solis Biodyne 5× HOT FIREPol Blend Master mix in a 20 µL reaction mixture, containing 1× Blend Master mix buffer (Solis Biodyne), 1.5 mM MgCl_2_, 200 µM of each deoxynucleoside triphosphates (dNTP) (Solis Biodyne), 25 pMol of each primer (BIOMERS, Germany), 2 unit of Hot FIREPol DNA polymerase (Solis Biodyne), Proofreading Enzyme, 5 µL of the extracted DNA, and sterile nuclease-free water was used to make up the reaction mixture.

Thermal cycling was conducted in a PTC 200 gradient thermal cycler for an initial denaturation for 15 min at 95°C followed by 35 amplification cycles of 30 s at 95°C, 1 min at 58°C and 1 min 30 s at 72°C. This was followed by a final extension step of 10 min at 72°C. The amplification product was separated on a 1.5% agarose gel, and electrophoresis was carried out at 80 V for 1 h 30 min. After electrophoresis, DNA bands were visualized by ethidium bromide staining. 100 bp DNA ladder was used as DNA molecular weight standard. An amplicon size of about 365 bp indicated the presence of *Bacillus* species.

### Statistical analysis

The study variables were presented as means (standard deviations) or frequencies and percentages. A multivariate logistic regression model was developed to determine the association between COVID-19 status (negative, asymptomatic, mild, and severe) and *Bacillus* species isolation status dichotomized into positive and negative. The model was adjusted for age, sex, and presence of comorbidity (dichotomized as present or not present). Statistical analysis was done using SPSS version 26.0, results were presented as adjusted odds ratio (AOR) with 95% confidence interval (95% CI), and statistical significance was set at a *P* value < 0.05.

## RESULTS

There were 450 study participants recruited into this study. Forty-three (9.6%) had invalid COVID-19 qRT-PCR and the information on the age and/or sex was missing for 19 (4.2%) participants. The complete data for 388 participants are presented in Table 1.

**TABLE 1:** Demographic characteristics, COVID-19 severity, and *Bacillus* isolation from the nasopharyngeal samples of adult Nigerians with COVID-19 (*N* = 388)

Demographic characteristics	Variable	Statistic
Sex	Female: *n* (%)	151 (38.9)
	Male: *n* (%)	237 (61.1)
Age	Mean (SD)	40.05 (13.563)
COVID-19	Negative *n* (%)	228 (58.8)
	Asymptomatic *n* (%)	28 (7.2)
	Mild *n* (%)	32 (8.2)
	Severe *n* (%)	100 (25.8)
Pre-existing condition	Yes *n* (%)	130 (33.5)
	No *n* (%)	258 (66.5)
*Bacillus* isolated	Yes *n* (%)	76 (19.6)
	No *n* (%)	312 (80.4%)

The age of participants ranged from 18 to 75 years with the mean age (standard deviation) age of 40.05 (13.563) years. Also, 237 (61.1%) participants were male, 228 (58.8%) had a negative COVID-19 test result, and 258 (66.5%) had no pre-existing medical condition. Among participants that reported having a pre-existing condition, 71 (18.3%) had high blood pressure and 22 (5.7%) had both type II diabetes mellitus and high blood pressure. There were *Bacillus* species isolated from 76 (19.6%) of the nasopharyngeal samples collected.

**Fig 1:**
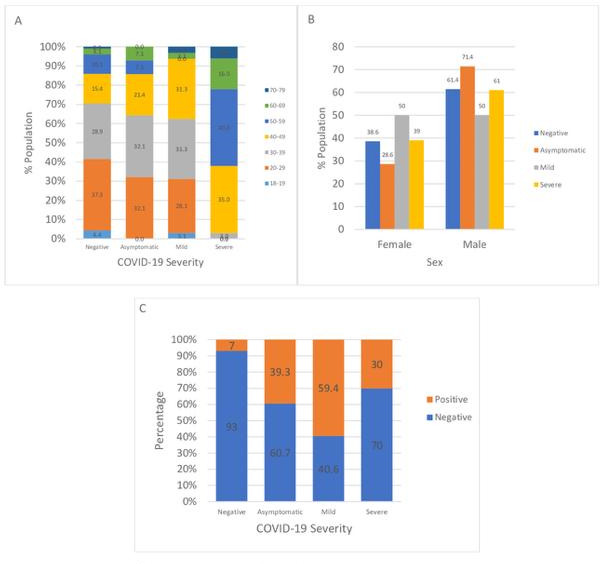
Association of the measured variables with COVID-19 severity. (**A**) Age distribution of participants vs COVID-19 severity. (**B**) Participant’s sex vs COVID-19 severity. (**C**) Nasal carriage of *Bacillus* species vs COVID-19 severity.

Figure 1 shows the association between each variable and COVID-19 severity. Chi square analysis showed an association between age and COVID-19 severity (*χ*^2^ = 150.792, *P* < 0.001), presence of a pre-existing condition and COVID-19 severity (*χ*^2^ = 198.511, *P* < 0.001), and the presence of *Bacillus* species and COVID-19 severity (*χ*^2^ = 68.815, *P* < 0.001). No association was seen between sex and COVID-19 (*χ*^2^ = 2.924, *P* = 0.403).

Table 2 shows that the presence of *Bacillus* species in the nasopharyngeal samples was associated with COVID-19 severity: patients with *Bacillus* species in the nasopharyngeal samples had significantly higher odds for severe COVID-19 than a negative COVID-19 result (AOR = 3.347, 95% CI: 1.359, 8.243). Also, patients with *Bacillus* species in the nasopharyngeal samples had significantly lower odds for severe COVID-19 than a mild COVID-19 result (AOR = 0.158, 95% CI: 0.055, 0.455).

**TABLE 2:** Associations between age, comorbidity, and the presence of nasal carriage of *Bacillus* species and COVID-19 severity (*N* = 388)^*a*^

	Severe vs negative		Severe vs asymptomatic		Severe vs mild	
Variable	AOR (95% CI)	*P*	AOR (95% CI)	*P*	AOR (95% CI)	*P*
Age	0.915 (0.886, 0.945)	<0.001*	0.929 (0.886, 0.975)	0.003*	0.891 (0.850, 0.934)	<0.001*
Sex	1.674 (0.829, 3.377)	0.151	1.147 (0.385, 3.415)	0.805	1.964 (0.739, 5.223)	0.176
Presence of a co-morbidity	0.087 (0.042, 0.182)	<0.001*	–	–	0.214 (0.070, 0.655)	0.007*
*Bacillus*	3.392 (1.377, 8.355)	0.008*	0.390 (0.124, 1.233)	0.109	0.165 (0.057, 0.473)	0.001*

^*a*^ AOR, adjusted odds ratio; CI, confidence interval; *, statistically significant at *P* < 0.05. The reference category is severe.

## DISCUSSION

The main objective of this study was to determine if there was an association between the nasal carriage of *Bacillus* species and COVID-19 severity, while controlling for other factors that have been reported to influence COVID-19 severity (21, 29). The other factors measured and controlled for in this study included age, sex, and the presence of a co-morbidity. In this study, age and co-morbidity were significantly associated with COVID-19. The association seen between age and the presence of co-morbidity and COVID-19 severity is not strange. Since the inception of the COVID-19 pandemic, these variables have been identified as having a strong association with COVID-19 severity, even in studies from Nigeria (21, 30, 31). However, though the proportion of males was higher than that of the females in this study, there was no significant association between sex and COVID-19 severity. Some studies in Nigeria have, nonetheless, reported a significant association between sex and COVID-19 severity, with males having the more severe form of COVID-19 (31–33). However, most of these studies did not assess if there was a significant difference in the prevalence of COVID-19 based on sex (32, 33).

The study findings showed a strong positive association between nasal carriage of *Bacillus* species and COVID-19 severity while participants who carried *Bacillus* species in their upper respiratory tract had a lower likelihood of developing severe COVID-19 when compared with mild cases. The study findings did not support the study hypothesis of a negative relationship between nasal carriage of *Bacillus* species and COVID-19 severity.

This is the first study showing a relationship between bacteria co-infection involving nasal carriage of *Bacillus* species and COVID-19. The *Bacillus* species isolated in this study can be classified as a co-infection as they were recovered from the participants at the point of SARS-COV-2 infection diagnosis (34). Our study results indicated that attention needs to be given to *Bacillus* species as a secondary bacterial co-infection in COVID-19 and a possible risk factor for severe COVID 19. Prior studies that evaluated respiratory bacteria co-infection in patients with COVID-19 identified *Acinetobacter baumannii*, *Staphylococcus aureus*, *Klebsiella pneumoniae,* and *Staphylococcus aureus* as the most isolated bacterial infection from respiratory tract cultures (35–37). We had also reported an association between the nasal carriage of *Moraxella catarrhalis* and *Chlamydophila pneumoniae* and COVID-19 (38).

The observed association between *Bacillus species*, a respiratory opportunistic pathogen, and the severity of COVID-19 may be connected to its association with the high risk for co-morbidities in patients with severe COVID-19. *Bacillus* counts are typically high in patients who are immunocompromised and patients with diabetes (39–42). These co-morbidities also increase the risk for severe COVID-19. Overall, attention has not been given to the possibility of *Bacillus* species being a respiratory opportunistic pathogen and its influence on COVID-19. With the exploration of *Bacillus* species in nasal sprays for the treatment of respiratory viral infections (17, 18), there is still a need to further identify (up to strain level) the *Bacillus* species isolated in this study and characterize the isolates by antibiotic susceptibility and virulence to better understand the properties of the isolates that may influence the severity of COVID-19.

We also found that individuals with *Bacillus* species in their upper respiratory tract may have a lower likelihood of developing severe COVID-19 when compared with mild cases. This finding is similar to the outcome of a prior study that demonstrated that treatment with lactobacilli had the potential to lower the viral load of SARS-CoV-2 in the upper respiratory tract without necessarily resolving the symptoms of COVID-19 (19). The implication of this is that the patient is still positive for SARS-CoV-2 testing, and the lowered SARS-CoV-2 viral load would also imply a mild COVID-19.

The lower likelihood of severe COVID-19 compared to mild COVID-19 when *Bacillus* is present may also be related to the reduction or improvement of the symptoms of SARS-CoV-2 infection by the *Bacillus* species. Metabolites, such as surfactins, iturin A, and fengycins, produced by some *Bacillus* species have antibacterial and antifungal activities (43–46). Surfactins have been shown to have an amphiphilic structure which helps modify surface hydrophobicity (47). This surface hydrophobicity is one of the properties of pulmonary surfactants which helps them inhibit the entry of respiratory viruses into the epithelial cells (15, 16, 48). The production of surfactins and other metabolites by *Bacillus* species may be responsible in easing the symptoms of COVID-19 and preventing the progression of the severity of the disease in mild cases of COVID-19. Again, there is a need to characterize the *Bacillus* species with regard to the production of metabolites that may influence its association with COVID-19 as seen in this study.

This study has its limitations. The cross-sectional study design used limits the generalizability of the results and can only suggest an association and not a cause-effect relationship. The use of nasopharyngeal respiratory tract cultures also limits the interpretation of this findings as upper respiratory tract samples are more subject to contamination and commensal pathogens, and thus, bacterial isolation from such specimen is often higher than those from blood cultures (35). We could not collect samples from the lower respiratory tract of the participants in this study as most of the participants did not have a productive cough. However, the collection of samples at the time of COVDI-19 diagnosis strengthens this study by showing that the isolated *Bacillus* species occur as a co-infection and not an acquired secondary bacterial infection. There is a need for further study to determine the pathogenicity and production of biosurfactants by the *Bacillus* species and the role this plays in COVID-19 severity. The use of lower respiratory tract specimen and/or blood culture should also be explored to further validate the association between *Bacillus* species co-infection and COVID-19 severity.

### Conclusion

This study suggests that the presence of *Bacillus* species in the nasopharyngeal tract could significantly influence the clinical progression or severity of COVID-19. This was a cross-sectional study, so it can only suggest a link between *Bacillus* species and the severity of COVID-19. However, because samples were taken at the time of diagnosis, COVID-19 patients should be screened for bacterial co-infection. This study also showed that the chance of getting severe COVID-19 is lower than the chance of getting mild COVID-19 when *Bacillus* is present. This suggests that *Bacillus* species could be useful in managing viral respiratory tract infections. More research is, however, required in this regard.
